# The Impact of 24 h Urinary Potassium Excretion on High-Density Lipoprotein Cholesterol and Chronic Disease Risk in Chinese Adults: A Health Promotion Study

**DOI:** 10.3390/nu16193286

**Published:** 2024-09-28

**Authors:** Xiaofu Du, Xiangyu Chen, Jie Zhang, Feng Lu, Chunxiao Xu, Jieming Zhong

**Affiliations:** Department of Chronic Disease Prevention and Control, Zhejiang Provincial Center for Disease Control and Prevention, No. 3399 Binsheng Road, Hangzhou 310051, China; xfdu@cdc.zj.cn (X.D.); xychen@cdc.zj.cn (X.C.); jiezhang@cdc.zj.cn (J.Z.); flu@cdc.zj.cn (F.L.); chxxu@cdc.zj.cn (C.X.)

**Keywords:** hypertension, diabetes mellitus, dyslipidemia, urinary sodium and potassium excretion, chronic diseases, comorbidity

## Abstract

Background: Research into the pivotal role of potassium in chronic diseases and their comorbidities remains scarce. Our aim is to elucidate the relationship between potassium and chronic diseases, including comorbid conditions, and to provide evidence-based recommendations for potassium intake in patients. Methods: This study is anchored in a representative, population-based survey conducted in Zhejiang Province, China, in 2017, encompassing participants aged 18 to 69 years. Data collection included questionnaire responses, physical measurements, and biological samples, obtained through a multistage cluster random sampling method. A subset of 1496 participants provided complete 24 h urine samples. Results: The median age of the participants was 48.0 years (interquartile range [IQR] 24.0), with 51.1% being female, and hypertension was identified in more than one third (35.6%) of the participants. The prevalence of diabetes was approximately 9.0%, dyslipidemia was found in 34.2%, and microalbuminuria in 8.8%. The 24 h urinary excretion levels were 3613.3 mg/24 h (IQR 2161.7) for sodium and 1366.0 mg/24 h (IQR 824.9) for potassium, respectively. Potassium excretion exhibited an inverse relationship with blood pressure. Furthermore, a positive correlation was observed between potassium excretion and high-density lipoprotein cholesterol (HDL-C) levels, with an elevation of 0.03 mmol/L (95% confidence interval [CI] 0.00 to 0.05). In binary logistic regression analysis, individuals in the fourth quartile of potassium excretion (Q4) exhibited an odds ratio (OR) of 0.56 (95% CI 0.36–0.87) for hypertension compared to those in the first quartile (Q1). Urinary potassium excretion was inversely associated with low HDL-C levels, with Q4 individuals having 0.62 times the odds of having low HDL-C levels (OR, 0.62; 95% CI 0.39–1.00) compared to Q1. Conclusions: Potassium excretion demonstrated a direct negative correlation with certain comorbidities. This study underscores the pivotal role of potassium in the management of chronic diseases and associated comorbidities, thereby highlighting the significance of potassium in both public health initiatives and clinical practice.

## 1. Introduction

The causal link between a high-salt diet and the prevalence of cardiovascular and cerebrovascular diseases, including hypertension and stroke, which contribute to a reduction in life-adjusted years of health, is well-established [1]. Despite a noticeable decrease in salt consumption in China to the current average of less than 10.0 g, it still exceeds the World Health Organization (WHO)’s recommended maximum of 5 g per day. Additionally, potassium intake falls short of the WHO’s guideline of more than 3.5 g per day [2]. Introducing additional measures to decrease sodium consumption and boost potassium intake is essential in the battle against hypertension and related cardiovascular conditions [3,4].

A substantial body of research has delved into the link between sodium consumption and the onset of cardiovascular and cerebrovascular illnesses [5,6]. However, the direct correlation between potassium and common chronic diseases warrants further investigation. Potassium, an indispensable electrolyte, is vital for human physiology and has significant implications for the well-being of the cardiovascular and cerebrovascular systems [7]. Research indicates that substituting regular salt with low-sodium salt can reduce blood pressure and potentially decrease the risk of all-cause mortality, as well as morbidity and mortality from cardiovascular and cerebrovascular diseases, including stroke [8]. Furthermore, several studies have highlighted the connection between hypokalemia (low blood potassium levels) and the incidence of stroke in patients with pre-existing cardiovascular and cerebrovascular conditions, hypertension, and diabetes [9,10]. The reduction in blood potassium levels appears to be associated with the occurrence and fatality of stroke [11].

In 2017, a cross-sectional survey was undertaken across Zhejiang Province with the aim of examining the relationship between urinary potassium levels and the occurrence of chronic diseases such as hypertension, diabetes, dyslipidemia, and microalbuminuria. The research also sought to explore the connection between potassium excretion and the co-occurrence of multiple health conditions. The findings from this study are expected to contribute to the development of more effective preventative and therapeutic approaches for chronic diseases, tackling their onset and further development.

## 2. Materials and Methods

### 2.1. Participant Recruitment

The research data originated from a 2017 cross-sectional survey in Zhejiang Province, China. Participants were recruited via a multistage, stratified random sampling approach. Only individuals aged 18 to 69, who had been continuously residing in the designated regions for at least 6 to 12 months, were considered eligible for the study. The survey encompassed 5 project sites across the province, comprising 2 rural and 3 urban regions, strategically located in and around the central part of Zhejiang Province. Villages or neighborhood committees, serving as the smallest recruitment units, compiled rosters to actively engage and enroll eligible respondents. In conclusion, the survey encompassed 7512 individuals who were representative of the province’s demographic, with 1496 of these participants providing comprehensive 24 h urine collections for evaluating sodium and potassium excretion. Details regarding the determination and selection of the sample size are extensively described in another section [12]. The study protocol was granted approval by the Ethics Review Committee of the Zhejiang Provincial Center for Disease Control and Prevention (CDC). All aspects of the project, including questionnaire surveys, physical measurements, and the collection and testing of biological samples, were conducted in strict compliance with pertinent guidelines and regulations.

### 2.2. Data Collection

Following comprehensive professional training, our team of investigators engaged in direct, face-to-face interviews to gather data. They administered a questionnaire addressing social demographic characteristics and behavioral habits, including smoking, alcohol consumption, and physical activity levels. Additionally, they conducted physical assessments to measure key indicators. The criteria for these related indicators have been clearly defined in our preceding research [13].

Systolic blood pressure (SBP) and diastolic blood pressure (DBP) were measured using internationally recognized protocols and quality control standards. Participants were seated for at least 5 min prior to the commencement of the measurements. Three successive readings were obtained with the participant in a seated position, ensuring a minimum interval of one minute between each reading. A validated automatic electronic sphygmomanometer (model HEM-7071, Omron Corp., Kyoto, Japan) was utilized, equipped with a cuff size appropriate for the participant’s arm circumference. The average of the three measurements was calculated to determine the participant’s blood pressure.

Participants were instructed to provide a venous blood sample from their arm. These biological samples were then transported under cold-chain conditions to the central laboratory at China Hangzhou KingMed Diagnostics Laboratory Inc. (Hangzhou, China), for the assessment of fasting plasma glucose (FPG) levels and a range of blood lipid measurements, which encompassed high-density lipoprotein cholesterol (HDL-C), low-density lipoprotein cholesterol (LDL-C), triglycerides (TG), and total cholesterol (TC).

A total of 1572 participants were requested to complete a single 24 h urine collection to evaluate their urinary sodium and potassium excretion levels. To encourage participants in the urine collection process, the project team supplied a concise guide and the essential collection barrels. They also provided a comprehensive explanation of the steps involved in the 24 h urine collection protocol [14]. During the collection, investigators recorded the start and end times and measured the volume of the urine. Urine collections were considered incomplete if they fell short of 22 h or exceeded 28 h in duration, if the volume was below 500 mL, or if the creatinine excretion varied by more than two standard deviations from the norm [15]. Refer to Supplemental Figure S1 for the participant flow chart.

### 2.3. The Definition of Chronic Diseases in This Study

In this study, we have established clear criteria for identifying common chronic diseases:

- Hypertension is identified when the mean SBP is 140 mmHg or higher and/or the mean DBP is 90 mmHg or higher. This diagnosis also applies to those with a documented history of hypertension who are receiving treatment with antihypertensive drugs [16].
- Diabetes is diagnosed in individuals who exhibit a FPG level of 7.0 mmol/L or higher, a random plasma glucose level of 11.1 mmol/L or higher, an oral glucose tolerance test result of 11.1 mmol/L or higher, a hemoglobin A1c level of 6.5% or higher, or those with a known history of type 2 diabetes who are undergoing treatment with hypoglycemic agents [17].
- Microalbuminuria is defined as a 24 h urinary albumin excretion that ranges from 30 to 300 mg within a 24 h period [18].
- Dyslipidemia is characterized by levels of TC of 6.2 mmol/L or higher, TG of 2.3 mmol/L or higher, LDL-C of 4.1 mmol/L or higher, and HDL-C below 1.0 mmol/L for males or below 1.3 mmol/L for females. It also includes individuals with a prior diagnosis of dyslipidemia who are undergoing lipid-lowering treatment [19].

Furthermore, this research investigates the distinct correlation between sodium and potassium urinary excretion rates and blood lipid concentrations, with low HDL-C being classified as levels below 1.0 mmol/L for males and below 1.3 mmol/L for females. These definitions are pivotal for the accurate classification and analysis of chronic disease prevalence within our study population [20].

Hypertension complicated with diabetes mellitus refers to participants who have both hypertension and diabetes mellitus, excluding other chronic diseases under consideration in this study. Similarly, hypertension complicated with dyslipidemia indicates that the respondents are affected by both hypertension and dyslipidemia, without the presence of other specified chronic conditions. Diabetes mellitus complicated with dyslipidemia signifies that the respondents have both diabetes and dyslipidemia as their only comorbidities. Lastly, hypertension complicated with both diabetes and dyslipidemia denotes the presence of all three chronic diseases concurrently.

### 2.4. Statistical Analysis

The study’s participant demographics were tabulated using percentages for discrete variables and averages with medians (along with interquartile range, IQR) for continuous data. The independent samples Mann–Whitney U test was employed to assess differences in demographic and health characteristics among various categories of sodium or potassium status. A multivariable linear regression model was applied to assess the relationships between 24 h urinary sodium and potassium output (for every 1000 mg increase) and the sodium-to-potassium ratio (for each 1-unit molar increase) with SBP, DBP, FPG, blood lipid profiles, and urinary microalbumin levels. Binary logistic regression was used to determine the correlation between the likelihood of chronic diseases or co-occurring conditions and higher quartiles (Q4 for the fourth, Q3 for the third, and Q2 for the second) of sodium or potassium excretion, with the first quartile (Q1) serving as the baseline. The analysis compared the adjusted odds ratios for disease presence versus absence. Statistical computations were executed using SPSS for Windows, version 26 (SPSS Inc., Chicago, IL, USA). A *p*-value below 0.05 was set as the threshold for statistical significance.

## 3. Results

### 3.1. Study Participants

The median age of the participants was 48.0 years (IQR 24.0), with 51.1% being female (Table 1). The median SBP and DBP values were recorded at 127.5 mmHg (IQR 24.3) and 78.3 mmHg (IQR 14.3), respectively. Significantly, hypertensive status was identified in more than a third of the participants, representing a prevalence rate of 35.6%. Additionally, the median FPG level was 4.9 mmol/L (IQR 0.8), and the prevalence of diabetes was approximately 9.0%. Dyslipidemia and microalbuminuria were observed in 34.2% and 8.8% of the participants, respectively. The examination of sodium and potassium excretion levels across various groups revealed notable disparities in demographic and lifestyle characteristics. These differences spanned age, gender, body mass index (BMI), ethnicity, geographical location, educational attainment, and habits related to smoking, alcohol consumption, and physical activity.

Among the participants initially designated for the 24 h urine collection, 1496 individuals, accounting for 95% of the 1572 initially selected, successfully submitted complete samples. Among the 1496 participants with complete urinary data, the 24 h urinary excretion levels were 3613.3 mg/24 h (IQR 2161.7) for sodium and 1366.0 mg/24 h (IQR 824.9) for potassium, respectively. The average excretion of urinary microalbumin was 4.8 mg/24 h (IQR 8.4). Additionally, there was a significant increase in urinary microalbumin levels among participants with elevated sodium excretion compared to those with lower levels (*p* < 0.05).

### 3.2. Associations between Urinary Sodium and Potassium Excretion and Biomarkers of Chronic Diseases

In the comprehensively adjusted linear regression analyses presented in Table 2, sodium excretion was positively correlated with SBP, increasing by 0.67 mmHg (95% Confidence Interval [CI] 0.10–1.25), and DBP, increasing by 0.42 mmHg (95% CI 0.07–0.77), per every extra 1000 mg of sodium consumed. In contrast, potassium excretion was inversely linked to SBP, showing a reduction of 2.77 mmHg (95% CI −4.14 to −1.40), and DBP, with a decrease of 0.88 mmHg (95% CI −1.73 to −0.04), for each additional 1000 mg of potassium ingested.

Moreover, a direct correlation was observed between the sodium-to-potassium molar ratio and SBP, corresponding to an elevation of 0.79 mmHg (95% CI 0.45–1.14), and DBP, with an increase of 0.32 mmHg (95% CI 0.11–0.54), for each unit increment in the ratio. Potassium excretion also demonstrated a positive association with HDL-C levels, marking an increment of 0.03 mmol/L (95% CI 0.00 to 0.05). Sodium excretion was also related to urinary microalbumin levels, with an increase of 1.09 mg (95% CI 0.18 to 2.01). The diagnostic results for all models were statistically significant, *p* < 0.001. The correlations between the diagnostic indices are presented in Supplemental Table S1.

### 3.3. Uriary Exception of Sodium and Potassium in Relation to Chronic Disease

Chronic disease status was treated as a binary variable in this analysis, necessitating the use of binary logistic regression analysis (Table 3).

In the comprehensively adjusted model, a notably inverse relationship was identified between urinary potassium excretion and the likelihood of hypertension. To be precise, participants in the uppermost quartile for potassium excretion (Q4) had a 56% lower likelihood of hypertension (odds ratio [OR], 0.56; 95% CI 0.36–0.87) when compared to those in the lowest quartile (Q1). Conversely, a higher sodium-to-potassium ratio among Q4 individuals corresponded to a 65% higher likelihood of hypertension (OR, 1.65; 95% CI 1.13–2.41) as opposed to Q1.

Simultaneously, urinary potassium excretion demonstrated a negative correlation with low HDL-C, with individuals in Q4 having 0.62 times the odds of having low HDL-C levels (OR, 0.62; 95% CI 0.39–1.00) compared to Q1. Conversely, there was a positive correlation between urinary sodium excretion and the levels of urinary microalbumin, with individuals in Q4 showing 1.95 times higher odds of having increased microalbumin levels (OR, 1.95; 95% CI 1.01–3.74) than those in Q1. The diagnostic results for all models were statistically significant, *p* < 0.001.

### 3.4. Uriary Exception of Sodium and Potassium in Relation to Comorbidity

We subsequently conducted an investigation into the direct association between sodium and potassium excretion levels and the presence of comorbidity (Table 4).

The findings revealed a notably inverse relationship between urinary potassium excretion and the co-occurrence of hypertension and dyslipidemia. Participants in Q4 of potassium excretion had 0.58 times the odds of this comorbidity (OR 0.58; 95% CI 0.34–0.98) compared to those in Q1. In contrast, a positive correlation was observed with the sodium-to-potassium ratio, where Q4 demonstrated 1.64 times the odds of comorbidity compared to Q1 (OR, 1.64; 95% CI 1.03–2.60). Urinary sodium excretion also showed a positive correlation with the comorbidity of diabetes mellitus and dyslipidemia, with Q4 having 2.67 times the odds of this comorbidity (OR, 2.67; 95% CI 1.00–7.20) when compared to Q1.

In the case of comorbidity encompassing hypertension, diabetes mellitus, and dyslipidemia, an elevated sodium-to-potassium ratio corresponded to a positive correlation. The odds ratios for Q3 and Q4 compared to Q1 were 3.27 (95% CI 1.14–9.35) and 2.91 (95% CI 1.00–8.48), respectively. The diagnostic results for all models were statistically significant, *p* < 0.001.

## 4. Discussion

The WHO advises a minimum daily potassium intake of 90 mmol per individual [21]. Yet, globally, including in China, the typical consumption levels are considerably below this benchmark, highlighting a significant public health concern [22]. Our study’s findings indicate that the low consumption of potassium and a high sodium-to-potassium ratio continue to be pressing health concerns in the region under investigation. Therefore, enhancing potassium consumption may be equally vital, and perhaps more readily achievable, than sodium reduction for the prevention and control of chronic diseases.

In general, the relationship between urinary sodium and potassium excretion is intricate and can be influenced by a multitude of factors, such as dietary habits, lifestyle, and the presence of other comorbidities. The urinary sodium-to-potassium ratio is increasingly recognized as a more reliable indicator of cardiovascular risk compared to the assessment of sodium or potassium excretion in isolation [23]. Research has indicated that cardiovascular disease risk can be substantially lowered through a reduction in sodium intake coupled with an increase in potassium consumption [24].

Recently, the health benefits of low-sodium salt have received significant scientific backing, particularly in nursing homes and rural areas of China [25]. In tandem with this, Chinese researchers have developed guidelines for the utilization and promotion of low-sodium salt. These guidelines aim to provide expert recommendations on the nationwide promotion and application of low-sodium salt [26]. Previous research has also established that a lower sodium-to-potassium ratio correlates with the use of salt-restriction measures [13].

Salt with reduced sodium content and increased potassium can make a substantial contribution to the prevention of cerebrovascular diseases. A comprehensive analysis of international clinical trials involving nearly 30,000 participants demonstrates that potassium salt, as a substitute for sodium salt, can lower blood pressure and offers broad cardiovascular protection across diverse populations [27]. A pivotal study in China indicates that nationwide efforts to substitute conventional salt with potassium-enriched salt could potentially avert approximately 500,000 deaths due to cardiovascular and cerebrovascular diseases each year [28]. One extensive study, conducted through long-term real-world observation, has further substantiated that substituting potassium salt for sodium salt can lead to a significant reduction in major cardiovascular and cerebrovascular events as well as all-cause mortality among high-risk cardiovascular disease populations, with no serious adverse events detected. Consequently, the adoption of salt substitution should be strongly advocated [29]. Of course, some studies have shown how it is important to recognize that while potassium is beneficial for cardiovascular and cerebrovascular health, hyperkalemia (elevated blood potassium levels) can present health dangers, such as arrhythmias, and in severe cases, may result in cardiac arrest. Thus, a balanced potassium intake is essential to prevent health risks.

While these studies provide valuable insights to a certain extent, they are not without contradictions. The discussions primarily revolve around the combined effect of reducing sodium and supplementing potassium through low-sodium salt, yet they fail to delineate the distinct roles of sodium and potassium individually.

The study indicates an association between higher potassium consumption and increased levels of HDL-C, which is recognized as the ‘good’ cholesterol that facilitates the clearance of LDL-C from the circulatory system. This link may be attributed to potassium’s function in managing blood pressure and maintaining fluid balance, thereby possibly impacting lipid metabolism and HDL-C concentrations [30,31]. Prior research has demonstrated a significant correlation between urinary sodium excretion and the presence of urinary albumin [18]. A meta-analysis has further indicated that a reduction in sodium intake can lead to a substantial decrease in albumin excretion. This effect is particularly notable in the context of treatments targeting the renin–angiotensin–aldosterone system blockade and among patients with renal impairment. Such findings are instrumental in elucidating the underlying mechanisms involved [32].

The issue of comorbidity in chronic diseases is highly meritorious of discussion. Currently, there is a relative dearth of studies that integrate sodium, potassium, and other factors with the concept of comorbidity. The INTERHEART study indicates that the presence of two or more risk factors within the same individual can amplify the risk of cardiovascular and cerebrovascular diseases to over 20 times that of hypertension in isolation [33]. Concurrently, obesity, excessive salt intake, diets low in potassium, and smoking are recognized as common risk factors across a spectrum of chronic diseases, which can interact in a direct manner. Thus, a comprehensive co-management approach to chronic diseases will be crucial in addressing future public health challenges. For instance, in the combined management of hypertension and dyslipidemia, the approach combines lifestyle modifications with pharmacological interventions, and statins can significantly mitigate the risk of cardiovascular diseases [34]. The guidelines issued by the European Society of Hypertension/European Society of Cardiology (ESH/ESC) and the European guidelines for dyslipidemia management exhibit some convergence in patient management strategies [35,36]. Restricting sodium intake, regular physical activity, and weight control, along with increasing the daily intake of potassium-rich food, are all effective strategies. Furthermore, incorporating dietary patterns like the low-salt DASH (Dietary Approaches to Stop Hypertension) diet and the calorie-restricted Mediterranean diet can be advantageous for individuals with hypertension and dyslipidemia [37,38]. This approach allows us to observe the regulatory effects of potassium on blood pressure and lipid profiles. Our findings align with this notion, demonstrating that patients with higher potassium intake exhibit a reduced likelihood of comorbidity, with those in Q4 having 0.58 times the odds compared to those in Q1.

Previous studies exploring the link between dietary potassium intake and the risk of developing diabetes mellitus have yielded inconsistent results [39,40]. Research indicates that the enhancement of insulin sensitivity attributed to the DASH diet is further amplified within the context of a more holistic lifestyle approach, encompassing regular exercise and weight management [41]. This study revealed that sodium and potassium excretion are not directly correlated with diabetes or the comorbidity of diabetes with hypertension. A community-based interventional study involving hypertensive and diabetic patients in rural China demonstrated that an extensive intervention program, including both medication and healthy lifestyle changes, did not result in a decrease in the occurrence of severe cardiovascular disease events during the 36-month study period [42]. The intervention included a diet rich in potassium, suggesting that the impact of dietary interventions may require a longer period of observation to fully manifest or to ensure the effectiveness of the intervention is optimally realized. A meta-analysis reveals a nonlinear dose–response relationship between dietary potassium intake and the risk of diabetes. The beneficial thresholds identified in select studies offer a reference for mitigating the risk of diabetes-related morbidity and mortality [43].

Primary health care initiatives, including screening and medication strategies for hypertension, diabetes, and proteinuria, have demonstrated favorable cost-effectiveness and positive health outcomes [44]. However, there is a research gap regarding the selection of healthy dietary practices, particularly those rich in potassium, for managing various comorbidities. This study’s findings indicate a negative correlation between potassium excretion and numerous comorbid conditions, thereby offering supportive evidence for lifestyle interventions as a therapeutic approach.

Our study boasts several clear strengths, primarily characterized by a rigorous survey design and an extensive population sample size, coupled with stringent quality control measures during data collection to minimize errors. Additionally, we collected 24 h urine samples from a subset of participants, utilizing this method to precisely assess sodium and potassium excretion—a task that is both challenging and complex. Furthermore, this study delves into prevalent chronic diseases and defines various types of co-morbidities, enriching the research content and lending significant public health implications to our findings [45].

Our study has inherent limitations. The omission of oral glucose tolerance tests in the identification of diabetes cases could have led to the underreporting of some instances, a limitation rooted in the project’s design and scope. Additionally, due to the logistical complexities and the requirement for substantial support, we confined our urine collection to a single 24 h cycle, which may have compromised the accuracy of our sodium and potassium excretion estimates [46,47]. One constraint of our research is the lack of capability to determine the estimated glomerular filtration rate (eGFR), a pivotal measure of kidney function, because blood creatinine levels were not recorded. Consequently, we could not determine the correlation between sodium and potassium excretion and eGFR levels [48]. Furthermore, due to the cross-sectional nature of this study, causality cannot be inferred, and there is a potential for selection bias and information bias within the findings.

## 5. Conclusions

The research suggests that potassium’s impact on reducing blood pressure is more significant, hinting that potassium supplementation could be a more potent approach for lowering blood pressure compared to sodium reduction. Additionally, there appears to be a positive link between elevated potassium excretion and increased HDL-C levels. Concurrently, a negative correlation exists between potassium excretion and the presence of dyslipidemia in conjunction with other comorbidities. This highlights the significance of acknowledging potassium’s role within the spectrum of chronic diseases and their co-occurring conditions, providing crucial insights and evidence to bolster non-pharmacological strategies for the control of chronic illnesses.

## Figures and Tables

**Table 1:** Characteristics of 1496 participants based on sodium and potassium status in a 2017 cross-sectional study in China.

Characteristic	All Subjects ^a^ (*n* = 1496)	Low Sodium (Less than 3613.3 mg/24 h, *n* = 745)	High Sodium (More than 3613.3 mg/24 h, *n* = 744)	*p* Value	Low Potassium (Less than 1366.0 mg/24 h, *n* = 749)	High Potassium (More than 1366.0 mg/24 h, *n* = 747)	*p* Value
Gender, *n* (%)				0.005 *			<0.001 *
Male	732 (48.9)	337 (45.2)	391 (52.6)		401 (53.5)	331 (44.3)	
Female	764 (51.1)	408 (54.8)	353 (47.4)		348 (46.5)	416 (55.7)	
Ethnicity, *n* (%)				0.65			0.025 *
Han	1476 (98.7)	734 (98.5)	735 (98.8)		734 (98.0)	742 (99.3)	
Others	20 (1.3)	11 (1.5)	9 (1.2)		15 (2.0)	5 (0.7)	
Household registration type, *n* (%)				0.77			0.004 *
Urban	655 (43.8)	328 (44.0)	322 (43.3)		300 (40.1)	355 (47.5)	
Rural	841 (56.2)	417 (56.0)	422 (56.7)		449 (59.9)	392 (52.5)	
Education, *n* (%)				0.009 *			<0.001 *
<9 years	486 (32.5)	261 (35.0)	224 (30.1)		282 (37.7)	204 (27.3)	
9–12 years	691 (46.2)	344 (46.2)	343 (46.1)		335 (44.7)	356 (47.7)	
>12 years	319 (21.3)	140 (18.8)	177 (23.8)		132 (17.6)	187 (25.0)	
Smoking status, *n* (%)				0.09			<0.001 *
Never smoked	1091 (72.9)	558 (74.9)	528 (71.0)		511 (68.2)	580 (77.6)	
Former smoker	68 (4.5)	32 (4.3)	36 (4.8)		34 (4.5)	34 (4.6)	
Current smoker	337 (22.5)	155 (20.8)	180 (24.2)		204 (27.2)	133 (17.8)	
Alcohol use status, *n* (%)	487 (32.6)	217 (29.1)	268 (36.0)	0.005 *	257 (34.3)	230 (30.8)	0.15
Physical activity, *n* (%)	610 (40.8)	295 (39.6)	313 (42.1)	0.33	267 (35.6)	343 (45.9)	<0.001 *
Stroke, *n* (%)	17 (1.1)	4 (0.5)	13 (1.7)	0.028 *	8 (1.1)	9 (1.2)	0.80
Coronary heart disease, *n* (%)	18 (1.2)	12 (1.6)	6 (0.8)	0.16	12 (1.6)	6 (0.8)	0.16
Self-report kidney disease, *n* (%)	7 (0.5)	6 (0.8)	1 (0.1)	0.06	3 (0.4)	4 (0.5)	0.70
Hypertension, *n* (%)	533 (35.6)	268 (36.0)	264 (35.5)	0.84	274 (36.6)	259 (34.7)	0.44
Diabete mellitus, *n* (%)	134 (9.0)	74 (9.9)	59 (7.9)	0.18	64 (8.5)	70 (9.4)	0.58
Dyslipidemia, *n* (%)	512 (34.2)	255 (34.2)	253 (34.0)	0.93	266 (35.5)	246 (32.9)	0.29
Microalbuminuria, *n* (%)	131 (8.8)	55 (7.4)	75 (10.1)	0.06	60 (8.0)	71 (9.5)	0.29
Antihypertensive medication use, *n* (%)	205 (13.7)	112 (15.0)	92 (12.4)	0.14	96 (12.8)	109 (14.6)	0.32
Age, year	48.0 (24.0)	49.0 (25.0)	46.0 (24.0)	<0.001 *	48.0 (25.0)	48.0 (24.0)	0.59
BMI, kg/m^2^	23.8 (4.5)	23.4 (4.3)	24.1 (4.5)	<0.001 *	23.6 (4.2)	24.0 (4.6)	0.003 *
SBP, mmHg	127.5 (24.3)	128.7 (24.7)	126.7 (24.7)	0.80	129.3 (23.2)	126.0 (25.9)	0.037 *
DBP, mmHg	78.3 (14.3)	78.0 (14.7)	79.0 (14.7)	0.014 *	78.3 (15.3)	78.3 (14.0)	0.82
FPG, mmol/L	4.9 (0.8)	4.9 (0.8)	4.9 (0.7)	0.63	4.9 (0.7)	4.9 (0.8)	0.49
HDL-C, mmol/L	1.3 (0.4)	1.3 (0.4)	1.3 (0.4)	0.52	1.2 (0.4)	1.3 (0.4)	0.012 *
LDL-C, mmol/L	2.7 (1.0)	2.7 (1.0)	2.7 (1.0)	0.34	2.7 (1.0)	2.7 (1.0)	0.06
TC, mmol/L	4.9 (1.2)	4.9 (1.2)	4.9 (1.2)	0.57	4.9 (1.2)	4.9 (1.2)	0.15
TG, mmol/L	1.2 (0.9)	1.2 (0.9)	1.2 (0.9)	0.21	1.2 (0.9)	1.2 (0.9)	0.15
24 h urinary sodium excretion, mg/24 h	3613.3 (2161.7)	2653.7 (1088.8)	4813.7 (1638.6)	<0.001 *	2941.2 (1819.5)	4348.7 (2112.4)	<0.001 *
24 h urinary potassium excretion, mg/24 h	1366.0 (824.9)	1127.5 (667.7)	1618.5 (848.3)	<0.001 *	1004.3 (391.3)	1827.7 (644.8)	<0.001 *
24 h urinary microalbumin, mg/24 h	4.8 (8.4)	3.8 (6.3)	5.7 (10.0)	<0.001 *	4.3 (7.5)	5.4 (9.9)	<0.001 *
24 h urinary creatinine, mg/24 h	1067.7 (635.4)	953.5 (595.2)	1182.8 (650.7)	<0.001 *	968.5 (599.7)	1162.2 (673.0)	<0.001 *
24 h urine volume, mL/24 h	1420.0 (620.0)	1270.0 (620.0)	1520.0 (570.0)	<0.001 *	1255.0 (610.0)	1530.0 (580.0)	<0.001 *

Samples sizes (n), median (IQR), and prevalence were unweighted. Independent sample Mann–Whitney u test was used to compare the characteristics of different sodium or potassium status. Classifications of ‘low’ and ‘high’ are based on the median values of sodium or potassium excretion. ^a^ Percentages are column percent. * *p* < 0.05. Abbreviations: BMI: body mass index; DBP: diastolic blood pressure; FPG: fasting plasma glucose; HDL-C: high-density lipoprotein cholesterol; LDL-C: low-density lipoprotein cholesterol; IQR: interquartile range; SBP: systolic blood pressure; TC: total cholesterol; TG: triglycerides.

**Table 2:** Association between 24 h urinary sodium and potassium excretion and their ratio with indicators of chronic diseases.

All Subjects, *n* = 1496	SBP	DBP	FPG	HDL-C	LDL-C	TC	TG	Urine Microalbumin
	β-Coefcient (95% CI) ^a^	β-Coefcient (95% CI) ^a^	β-Coefcient (95% CI) ^a^	β-Coefcient (95% CI) ^a^	β-Coefcient (95% CI) ^a^	β-Coefcient (95% CI) ^a^	β-Coefcient (95% CI) ^a^	β-Coefcient (95% CI) ^a^
**Sodium excretion**								
Adjusted for age, sex, ethnicity ^b^	1.15 * (0.55 to 1.75)	0.76 * (0.39 to 1.13)	0.00 (−0.04 to 0.05)	0.00 (−0.01 to 0.01)	0.02 (0.00 to 0.05)	0.02 (−0.01 to 0.05)	0.01 (−0.03 to 0.06)	1.08 * (0.17 to 2.00)
Fully adjusted model ^c^	0.67 * (0.10 to 1.25)	0.42 * (0.07 to 0.77)	−0.02 (−0.05 to 0.02)	0.00 (−0.01 to 0.02)	0.01 (−0.02 to 0.03)	0.00 (−0.03 to 0.04)	−0.02 (−0.06 to 0.03)	1.09 * (0.18 to 2.01)
**Potassium excretion**								
Adjusted for age, sex, ethnicity ^b^	−3.00 * (−4.42 to −1.58)	−0.99 * (−1.86 to −0.11)	0.00 (−0.11 to 0.11)	0.02 (0.00 to 0.05)	0.02 (−0.04 to 0.08)	0.02 (−0.05 to 0.10)	−0.10 (−0.21 to 0.01)	0.77 (−1.40 to 2.93)
Fully adjusted model ^c^	−2.77 * (−4.14 to −1.40)	−0.88 * (−1.73 to −0.04)	0.01 (−0.08 to 0.09)	0.03 * (0.00 to 0.05)	0.03 (−0.03 to 0.09)	0.05 (−0.03 to 0.13)	−0.07 (−0.18 to 0.05)	0.25 (−1.94 to 2.43)
**Sodium-to-potassium ratio**								
Adjusted for age, sex, ethnicity ^b^	0.90 * (0.54 to 1.26)	0.39 * (0.17 to 0.61)	0.00 (−0.03 to 0.03)	0.00 (−0.01 to 0.01)	0.00 (−0.02 to 0.01)	0.00 (−0.02 to 0.02)	0.03 (0.00 to 0.05)	0.21 (−0.34 to 0.77)
Fully adjusted model ^c^	0.79 * (0.45 to 1.14)	0.32 * (0.11 to 0.54)	−0.01 (−0.03 to 0.01)	0.00 (−0.01 to 0.01)	−0.01 (−0.02 to 0.01)	−0.01 (−0.03 to 0.01)	0.02 (−0.01 to 0.05)	0.33 (−0.23 to 0.88)

^a^ β-coefficients for sodium and potassium indicate the change in mmHg of blood pressure associated with per 1000 mg difference in excretion; β-coefficients for the ratio of sodium-to-potassium indicate the change in mmHg of blood pressure associated with each 1-unit increase in molar ratio. In addition, sodium intake was adjusted in the regression models for potassium, and vice versa, and the models for the sodium-to-potassium ratio did not adjust for sodium and potassium excretion. ^b^ Preliminary adjusted model including age, sex, and ethnicity. ^c^ Fully adjusted models included age, sex, and ethnicity plus body mass index, education level, history of cardiovascular disease, diabetes mellitus status, self-reported chronic kidney disease, antihypertensive medication use, smoking status, alcohol use status, and physical activity. * *p* < 0.05 forβ-coefficient in the regression model. Abbreviations: CI, confidence interval; DBP: diastolic blood pressure; FPG: fasting plasma glucose; HDL-C: high-density lipoprotein cholesterol; LDL-C: low-density lipoprotein cholesterol; SBP: systolic blood pressure; TC: total cholesterol; TG: triglycerides.

**Table 3:** Relationship of chronic diseases with 24 h urinary sodium and potassium excretion and their ratio among adults aged 18 to 69 in Zhejiang Province, 2017.

All Subjects, *n* = 1496	Q1	Q2	Q3	Q4
	OR, 95% CI	OR, 95% CI	OR, 95% CI	OR, 95% CI
**Hypertension ^a^**				
Sodium excretion, mg/24 h				
Adjusted for age, sex, ethnicity ^b^	1.00	0.99 (0.69–1.40)	1.10 (0.77–1.58)	1.53 * (1.03–2.27)
Fully adjusted model ^c^	1.00	0.88 (0.60–1.29)	1.02 (0.69–1.51)	1.24 (0.81–1.92)
Potassium excretion, mg/24 h				
Adjusted for age, sex, ethnicity ^b^	1.00	0.64 * (0.45–0.91)	0.79 (0.55–1.13)	0.62 * (0.42–0.92)
Fully adjusted model ^c^	1.00	0.57 * (0.39–0.84)	0.66 * (0.44–0.98)	0.56 * (0.36–0.87)
Sodium-to-potassium ratio				
Adjusted for age, sex, ethnicity ^b^	1.00	1.40 * (1.00–1.97)	1.37 (0.97–1.93)	1.74 * (1.23–2.45)
Fully adjusted model ^c^	1.00	1.18 (0.82–1.69)	1.24 (0.85–1.81)	1.65 * (1.13–2.41)
**Diabetes**				
Sodium excretion, mg/24 h				
Adjusted for age, sex, ethnicity ^b^	1.00	1.42 (0.84–2.41)	0.87 (0.49–1.56)	1.24 (0.67–2.29)
Fully adjusted model ^c^	1.00	1.38 (0.80–2.39)	0.79 (0.43–1.45)	1.00 (0.52–1.90)
Potassium excretion, mg/24 h				
Adjusted for age, sex, ethnicity ^b^	1.00	0.75 (0.43–1.30)	1.06 (0.62–1.83)	0.90 (0.50–1.63)
Fully adjusted model ^c^	1.00	0.79 (0.44–1.41)	1.13 (0.63–2.02)	0.99 (0.53–1.87)
Sodium-to-potassium ratio				
Adjusted for age, sex, ethnicity ^b^	1.00	1.70 * (1.01–2.86)	1.47 (0.85–2.53)	1.23 (0.70–2.16)
Fully adjusted model ^c^	1.00	1.44 (0.84–2.49)	1.32 (0.75–2.34)	1.06 (0.58–1.92)
**Dyslipidemia**				
Sodium excretion, mg/24 h				
Adjusted for age, sex, ethnicity ^b^	1.00	1.16 (0.84–1.61)	0.92 (0.66–1.29)	1.39 (0.97–1.99)
Fully adjusted model ^c^	1.00	1.11 (0.79–1.56)	0.83 (0.58–1.18)	1.10 (0.76–1.61)
Potassium excretion, mg/24 h				
Adjusted for age, sex, ethnicity ^b^	1.00	1.02 (0.74–1.40)	1.09 (0.78–1.52)	0.68 * (0.47–0.97)
Fully adjusted model ^c^	1.00	1.08 (0.77–1.50)	1.12 (0.79–1.60)	0.74 (0.50–1.10)
Sodium-to-potassium ratio				
Adjusted for age, sex, ethnicity ^b^	1.00	1.41 * (1.03–1.93)	1.26 (0.92–1.73)	1.29 (0.94–1.77)
Fully adjusted model ^c^	1.00	1.25 (0.90–1.73)	1.12 (0.80–1.55)	1.05 (0.75–1.48)
**Low HDL-C**				
Sodium excretion, mg/24 h				
Adjusted for age, sex, ethnicity ^b^	1.00	1.08 (0.75–1.57)	0.76 (0.51–1.13)	1.21 (0.80–1.82)
Fully adjusted model ^c^	1.00	1.06 (0.72–1.56)	0.72 (0.48–1.08)	1.04 (0.67–1.59)
Potassium excretion, mg/24 h				
Adjusted for age, sex, ethnicity ^b^	1.00	1.15 (0.80–1.66)	1.29 (0.88–1.89)	0.64 * (0.41–0.98)
Fully adjusted model ^c^	1.00	1.15 (0.79–1.69)	1.25 (0.84–1.87)	0.62 * (0.39–1.00)
Sodium-to-potassium ratio				
Adjusted for age, sex, ethnicity ^b^	1.00	1.23 (0.85–1.79)	1.09 (0.75–1.58)	1.35 (0.94–1.94)
Fully adjusted model ^c^	1.00	1.12 (0.76–1.65)	1.03 (0.70–1.53)	1.22 (0.83–1.79)
**Microalbuminuria**				
Sodium excretion, mg/24 h				
Adjusted for age, sex, ethnicity ^b^	1.00	1.57 (0.87–2.82)	1.54 (0.85–2.80)	2.11 * (1.13–3.92)
Fully adjusted model ^c^	1.00	1.47 (0.80–2.70)	1.58 (0.85–2.93)	1.95 * (1.01–3.74)
Potassium excretion, mg/24 h				
Adjusted for age, sex, ethnicity ^b^	1.00	1.07 (0.62–1.87)	1.10 (0.62–1.95)	1.00 (0.55–1.83)
Fully adjusted model ^c^	1.00	1.09 (0.61–1.95)	1.10 (0.60–1.99)	1.01 (0.53–1.92)
Sodium-to-potassium ratio				
Adjusted for age, sex, ethnicity ^b^	1.00	0.83 (0.49–1.42)	1.44 (0.88–2.33)	0.79 (0.46–1.36)
Fully adjusted model ^c^	1.00	0.66 (0.38–1.17)	1.32 (0.80–2.19)	0.70 (0.39–1.24)

^a^ Chronic disease status was a binary variable and binary logistic regression analysis was performed. ^b^ Preliminary adjusted model including age, sex, and ethnicity. ^c^ Fully adjusted models included age, sex, and ethnicity plus body mass index, education level, history of cardiovascular disease, diabetes mellitus status, self-reported chronic kidney disease, antihypertensive medication use, smoking status, alcohol use status, and physical activity. * *p* < 0.05. Abbreviations: CI, confidence interval; HDL-C: high-density lipoprotein cholesterol; OR: odds ratio; Q1: the first quartile; Q2: the second quartile; Q3: the third quartile; Q4: the fourth quartile.

**Table 4:** Relationship of comorbidity of chronic diseases with 24 h urinary sodium and potassium excretion and their ratio among adults aged 18 to 69 in Zhejiang Province, 2017.

All Subjects, *n* = 1496	Q1	Q2	Q3	Q4
	OR, 95% CI	OR, 95% CI	OR, 95% CI	OR, 95% CI
**Hypertension complicated with diabetes mellitus ^a^**				
Sodium excretion, mg/24 h				
Adjusted for age, sex, ethnicity ^b^	1.00	1.21 (0.66–2.23)	0.87 (0.45–1.68)	1.36 (0.68–2.73)
Fully adjusted model ^c^	1.00	1.14 (0.60–2.19)	0.76 (0.37–1.55)	1.09 (0.51–2.31)
Potassium excretion, mg/24 h				
Adjusted for age, sex, ethnicity ^b^	1.00	0.72 (0.38–1.36)	1.01 (0.54–1.89)	0.73 (0.37–1.47)
Fully adjusted model ^c^	1.00	0.59 (0.30–1.17)	0.87 (0.44–1.71)	0.70 (0.33–1.47)
Sodium-to-potassium ratio				
Adjusted for age, sex, ethnicity ^b^	1.00	1.58 (0.85–2.94)	1.64 (0.87–3.08)	1.43 (0.75–2.73)
Fully adjusted model ^c^	1.00	1.24 (0.65–2.39)	1.32 (0.68–2.59)	1.33 (0.67–2.63)
**Hypertension complicated with dyslipidemia**				
Sodium excretion, mg/24 h				
Adjusted for age, sex, ethnicity ^b^	1.00	1.05 (0.69–1.60)	1.11 (0.73–1.71)	1.50 (0.94–2.38)
Fully adjusted model ^c^	1.00	0.93 (0.59–1.46)	0.90 (0.57–1.43)	1.04 (0.63–1.73)
Potassium excretion, mg/24 h				
Adjusted for age, sex, ethnicity ^b^	1.00	0.85 (0.57–1.28)	0.91 (0.60–1.39)	0.58 * (0.36–0.94)
Fully adjusted model ^c^	1.00	0.81 (0.52–1.27)	0.83 (0.52–1.32)	0.58 * (0.34–0.98)
Sodium-to-potassium ratio				
Adjusted for age, sex, ethnicity ^b^	1.00	1.63 * (1.07–2.49)	1.65 * (1.07–2.54)	1.88 * (1.23–2.88)
Fully adjusted model ^c^	1.00	1.37 (0.87–2.15)	1.42 (0.90–2.25)	1.64 * (1.03–2.60)
**Diabetes mellitus complicated with dyslipidemia**				
Sodium excretion, mg/24 h				
Adjusted for age, sex, ethnicity ^b^	1.00	2.31 (0.95–5.65)	1.79 (0.70–4.57)	3.58 * (1.41–9.09)
Fully adjusted model ^c^	1.00	2.32 (0.92–5.89)	1.39 (0.52–3.72)	2.67 * (1.00–7.20)
Potassium excretion, mg/24 h				
Adjusted for age, sex, ethnicity ^b^	1.00	0.95 (0.42–2.17)	1.24 (0.55–2.79)	1.00 (0.42–2.35)
Fully adjusted model ^c^	1.00	1.08 (0.45–2.58)	1.37 (0.57–3.28)	1.17 (0.46–2.95)
Sodium-to-potassium ratio				
Adjusted for age, sex, ethnicity ^b^	1.00	2.20 (0.93–5.20)	2.60 * (1.11–6.09)	2.41 * (1.02–5.67)
Fully adjusted model ^c^	1.00	1.65 (0.67–4.08)	2.27 (0.94–5.46)	1.99 (0.81–4.88)
**Hypertension complicated with diabetes and dyslipidemia**				
Sodium excretion, mg/24 h				
Adjusted for age, sex, ethnicity ^b^	1.00	1.65 (0.64–4.28)	1.44 (0.54–3.86)	3.04 * (1.14–8.12)
Fully adjusted model ^c^	1.00	1.52 (0.56–4.13)	1.03 (0.36–2.93)	2.17 (0.76–6.20)
Potassium excretion, mg/24 h				
Adjusted for age, sex, ethnicity ^b^	1.00	1.13 (0.47–2.74)	1.27 (0.52–3.13)	0.76 (0.28–2.07)
Fully adjusted model ^c^	1.00	1.01 (0.39–2.58)	1.19 (0.45–3.15)	0.80 (0.28–2.32)
Sodium-to-potassium ratio				
Adjusted for age, sex, ethnicity ^b^	1.00	2.26 (0.77–6.65)	3.82 * (1.38–10.62)	3.27 * (1.16–9.20)
Fully adjusted model ^c^	1.00	1.68 (0.55–5.21)	3.27 * (1.14–9.35)	2.91 * (1.00–8.48)

^a^ Comorbidity of chronic diseases status was a binary variable and binary logistic regression analysis was performed. ^b^ Preliminary adjusted model including age, sex, and ethnicity. ^c^ Fully adjusted models included age, sex, and ethnicity plus body mass index, education level, history of cardiovascular disease, diabetes mellitus status, self-reported chronic kidney disease, antihypertensive medication use, smoking status, alcohol use status, and physical activity. * *p* < 0.05. Abbreviations: CI, confidence interval; OR: odds ratio; Q1: the first quartile; Q2: the second quartile; Q3: the third quartile; Q4: the fourth quartile.

## Data Availability

The data presented in this study are available on request from the corresponding author. The data are not publicly available due to the project in progress and privacy.

## Supplementary Materials

The following supporting information can be downloaded at https://www.mdpi.com/article/10.3390/nu16193286/s1, Figure S1: Participant flow chart; Table S1: Correlation analysis of diagnostic indicators.

## Abbreviations

BMI: body mass index; BP: blood pressure; CDC: Center for Disease Control and Prevention; CI, confidence interval; CVD: cardiovascular diseases; DASH: Dietary Approaches to Stop Hypertension; DBP: diastolic blood pressure; eGFR: estimated glomerular filtration rate; FPG: fasting plasma glucose; HDL-C: high-density lipoprotein cholesterol; LDL-C: low-density lipoprotein cholesterol; OR: odds ratio; IQR: interquartile range; Q1: the first quartile; Q2: the second quartile; Q3: the third quartile; Q4: the fourth quartile; SBP: systolic blood pressure; TC: total cholesterol; TG: triglycerides; WHO: World Health Organization.
