# Supplementary material for: The Impact of 24 h Urinary Potassium Excretion on High-Density Lipoprotein Cholesterol and Chronic Disease Risk in Chinese Adults: A Health Promotion Study
Source: Nutrients. 2024 Sep 28;16(19):3286. doi: 10.3390/nu16193286 (PMC11478645; doi:10.3390/nu16193286)
Supplement: Supplementary file 1 [file nutrients-16-03286-s001.zip › nutrients-3214102-supplementary.pdf]

Supplementary Materials

The Impact of 24 h Urinary Potassium Excretion on High-Density Lipoprotein Cholesterol and Chronic Disease Risk in Chinese Adults: A Health Promotion Study

Xiaofu Du, Xiangyu Chen, Jie Zhang, Feng Lu, Chunxiao Xu and Jieming Zhong *

Department of Chronic Disease Prevention and Control, Zhejiang Provincial Center for Disease Control and Prevention, No. 3399 Binsheng Road, Hangzhou 310051, China; xfdu@cdc.zj.cn (X.D.);
xychen@cdc.zj.cn (X.C.); jiezhang@cdc.zj.cn (J.Z.); flu@cdc.zj.cn (F.L.); chxxu@cdc.zj.cn (C.X.)
* Correspondence: jmzhong@cdc.zj.cn

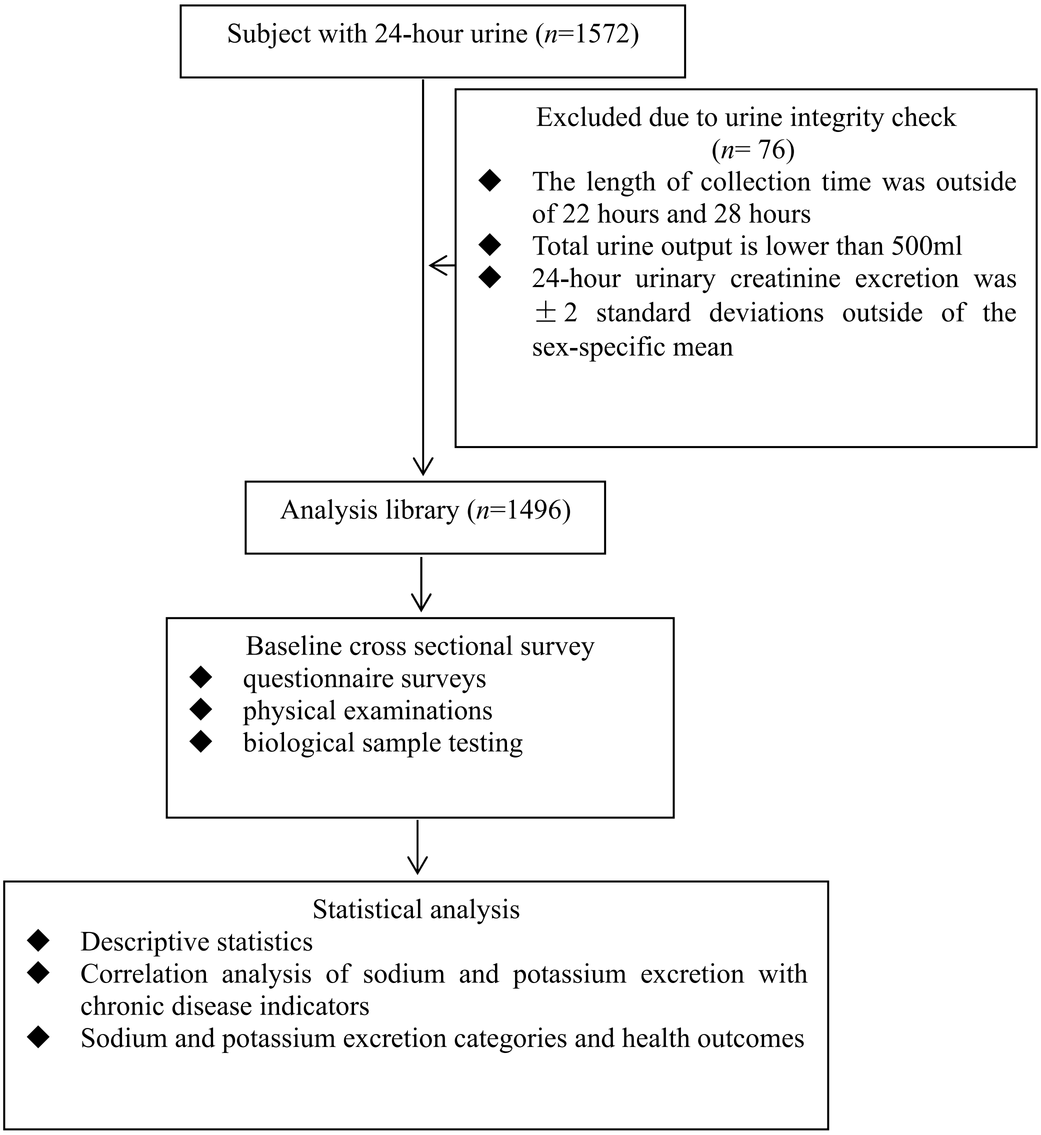

Supplemental Figure S1. Participant flow chart.

Supplemental Table S1. Correlation analysis of diagnostic indicators

	SBP	DBP	FPG	HDL-C	LDL-C	TC	TG	Urine microalbumin	Sodium excretion	Potassium excretion	Sodium-to-potassium ratio
SBP	1										
DBP	0.75**	1									
FPG	0.27**	0.17**	1								
HDL-C	-0.12**	-0.16**	-0.03	1							
LDL-C	0.17**	0.18**	0.10**	-0.01	1						
TC	0.21**	0.21**	0.16**	0.23**	0.82**	1					
TG	0.21**	0.24**	0.17**	-0.36**	0.01	0.36**	1				
Urine microalbumin	0.12**	0.12**	0.09**	-0.04	0.02	0.05	0.08**	1			
Sodium excretion	-0.01	0.06*	-0.03	-0.01	0.04	0.01	-0.01	0.08**	1		
Potassium excretion	-0.09**	-0.02	-0.01	0.06*	0.04	0.03	-0.05	0.05	0.48**	1	
Sodium-to-potassium ratio	0.10**	0.09**	-0.02	-0.04	-0.02	-0.03	0.06*	0.03	0.40**	-0.45**	1

*$P < 0.05$ and **$P < 0.001$ indicate significant correlations for r in the Pearson analysis. Abbreviations: DBP: diastolic blood pressure; FPG: fasting plasma glucose; HDL-C: high-density lipoprotein cholesterol; LDL-C: low-density lipoprotein cholesterol; SBP: systolic blood pressure; TC: total cholesterol; TG: triglycerides.
